# Inhibition of FXIIa attenuates kidney fibrosis in mice with unilateral ureteral obstruction

**DOI:** 10.1007/s00018-025-05988-z

**Published:** 2025-11-29

**Authors:** Daniel Kalina, Daniel P. Potaczek, Jinyang Zeng-Brouwers, Mario Boehm, Marc W. Nolte, Maximilian Bielohuby, Ralph T. Schermuly, Liliana Schaefer, Malgorzata Wygrecka

**Affiliations:** 1 CSL Innovation GmbH, Marburg, Germany; 2 Center for Infection and Genomics of the Lung (CIGL), Faculty of Medicine, Justus Liebig University (JLU), Universities of Giessen and Marburg Lung Center, Aulweg 132, 35392 Giessen, Germany; 3 Translational Inflammation Research Division & Core Facility for Single Cell Multiomics, Medical Faculty, Philipps-University Marburg, Marburg, Germany; 4 Institute of Pharmacology and Toxicology, Goethe University, Frankfurt, Germany; 5 Department of Internal Medicine, Member of the German Center for Lung Research, Justus Liebig University of Giessen, Giessen, Germany; 6 Institute for Lung Health (ILH), Justus Liebig University (JLU), Giessen, Germany

**Keywords:** Factor XII, Senescence, Epithelial cells, Kidney fibrosis

## Abstract

Kidney fibrosis is a common manifestation of chronic kidney diseases, with parenchymal tissue scarring serving as a histologic predictor of functional deterioration. Considering the relationships between contact-phase system activation and renal fibrosis as well as potential direct profibrotic activities of factor XII (FXII), we hypothesized that FXII inhibition with an anti-FXII/activated FXII (FXIIa) antibody (3F7) demonstrates therapeutic efficacy in a mouse model of unilateral ureteral obstruction (UUO). Treatment of UUO mice with 3F7 attenuated kidney fibrosis, as evidenced by preserved tissue structure, decreased deposition of collagen, and diminished apoptosis, but increased proliferation of tubular epithelial cells. No effect was observed with the administration of C1 esterase inhibitor, which serves as a primary plasma inhibitor of FXIIa and kallikrein. Transcriptome analysis revealed that 3F7 therapy predominantly affects stress-activated protein kinase signaling cascades and signal transduction in response to DNA damage. Exposure of renal epithelial cells to FXII or FXIIa triggered p21 expression in an Akt- and ERK1/2-dependent manner. Accordingly, treatment of UUO mice with 3F7 reduced numbers of p21^+^ renal tubular epithelial cells. Our work provides proof-of-concept data supporting efficacy of 3F7 in the UUO model and unravels molecular mechanisms underlying the protective role of FXII inhibition in chronic kidney injury.

**Supplementary Information:**

The online version contains supplementary material available at 10.1007/s00018-025-05988-z.

## Introduction

More than 1 in 7 US adults (about 35.5 million people) are estimated to suffer from chronic kidney disease (CKD) [1]. This prevalence, together with a lack of appropriate curative treatments, calls for the development of novel therapeutic strategies for CKD [2]. Renal fibrosis is considered to be the final manifestation of most CKDs, independently of their primary etiology [2, 3].

Nearly 99% of the kidney parenchyma consists of tubulointerstitium. The loss of tubular epithelial cells (TECs), caused by enhanced cell death and/or decreased proliferation/regeneration, represents the initial and decisive event responsible for driving renal diseases towards fibrosis [3–5]. Following injury, TECs undergo apoptosis and necrosis, or various adaptive changes, including metabolic shifts, as well as epigenetic and transcriptional remodeling, to orchestrate coagulation, inflammation, fibroblast activation and subsequent tissue regeneration [6]. These originally adaptive alterations may become maladaptive and contribute to the development and progression of renal fibrosis [6, 7]. Thus, understanding the molecular mechanisms driving the transition from adaptive to maladaptive kidney tissue remodeling, partially manifested by a controlled TEC phenotypic switch or cell death followed by proliferation and regeneration, is crucial for restoring kidney function [7, 8].

Remarkably, patients with CKD frequently exhibit a hypercoagulable state that increases the risk of thrombotic complications. This condition results from a complex interplay of factors, including endothelial cell dysfunction, inflammation, and oxidative stress, which may ultimately lead to fibroblast activation and fibrogenesis [9, 10]. The contribution of coagulation to the development and progression of renal pathologies is further supported by evidence that patients carrying the factor V Leiden mutation are predisposed to delayed graft function, acute rejection episodes, and chronic graft dysfunction following kidney transplantation [11, 12]. Despite these observations, the role of factor XII (FXII)—a key mediator at the intersection of coagulation and inflammation—remains poorly characterized in renal diseases.

FXII is mainly produced by hepatocytes and secreted into the bloodstream as a single-chain zymogen [13]. Upon conversion to a two-chain protease, activated FXII (FXIIa) initiates the intrinsic blood coagulation pathway [13], converts prekallikrein to kallikrein, which in turn liberates bradykinin (BK) from high molecular weight kininogen, and activates a classical complement pathway [14]. Kallikrein-kinin-mediated signaling and the complement system have been described to impact renal inflammation and fibrosis in preclinical models and patients [15]. Especially, tubular inflammation was linked to BK-induced MAPK signaling and both receptors, B1KR and B2KR, are discussed as potential therapeutic targets to prevent kidney injury and tubulointerstitial fibrosis [16]. While overall complement system activation was associated with accelerated senescence during ischemia–reperfusion injury due to reactive oxygen species generation [17], specifically complement component 3 and 5a have been described to exacerbate tubulointerstitial fibrosis by promoting M1 macrophage polarization and fibroblast proliferation [18]. FXII itself was found to regulate proliferation of epithelial cells, fibroblasts, hepatocytes and endothelial cells [13, 19–21], the latter through the activation of the ERK1/2/PI3K signaling pathway following binding to the urokinase receptor (uPAR) and complex formation with β1 integrin and epidermal growth factor receptor (EGFR) [20, 21].

Recently, FXII-uPAR-β1 integrin-induced senescence of kidney TECs in diabetic kidney disease (DKD) was described suggesting a function of FXII in the development of CKD [15]. Based on this observation and previous findings demonstrating the role of FXII in the regulation of inflammatory and cellular processes [19, 22], we hypothesize that inhibition of FXIIa provides protection against kidney fibrosis induced by unilateral ureteral obstruction (UUO) [23].

## Methods

### UUO model

The unilateral ureteral obstruction model was induced in 10- to 12-week-old male C57BL/6 J mice under ketamine/xylazine-induced anesthesia (50 mg/5 mg/kg). After a left flank incision, the ureter was exposed and double ligated using 5–0 silk, followed by closure of the incision under aseptic conditions. Mice were divided into two groups: one received an IgG control antibody (MuBM4-MuG1K; 1 mg per mouse; CSL Innovation GmbH, Marburg, Germany), and the other received recombinant fully human antibody 3F7 (1 mg per mouse; CSL Innovation GmbH). 3F7 preferentially binds to activated forms of FXII, as compared to zymogen FXII and it inhibits the FXIIa protease activity at an IC50 (half maximal inhibitory concentration) of 13 nM [24]. Single-dose pharmacokinetic and pharmacodynamic studies with 3F7 at 30 mg/kg in mice, including the i.v. administration route, support sufficient antibody levels for a robust FXIIa inhibition over the complete treatment period in the performed UUO model (data not shown). In other experiments, mice received either C1 esterase inhibitor Berinert® (600U per mouse, diluted in saline) or saline. The substances were injected intravenously in the tail vein immediately after ligation and then daily thereafter until the mice were sacrificed for analysis. The contralateral kidney served as healthy control. Plasma and kidneys were analyzed on day 3 and 10 post UUO. Briefly, blood was collected into prefilled EDTA tubes, centrifuged at 1500 g for 10 min at 4 °C, aliquoted and stored at -80 °C. Kidneys were harvested and either snap frozen and stored at -80 °C for further analysis or fixed in 4% paraformaldehyde (PFA). Mice were housed at room temperature in an environmentally controlled SPF animal facility with a 12 h light/dark cycle and had access to a standard maintenance rodent diet (Altromin1324, Lage, Germany) and filtered tap water ad libitum.

### PAS staining and quantitative assessment of tubular injury

The kidney tissue blocks were cut into 3-µm-thick sections, which were then subjected to routine Periodic Acid-Schiff (PAS) staining (all reagents were purchased from Carl Roth, Karlsruhe, Germany). Tubular injury was scored semi-quantitatively by three blinded observers, who examined at least 20 cortical fields (× 200 magnification) of PAS-stained kidney sections. Tubular injury was defined as tubular dilation, tubular atrophy, tubular cast formation, sloughing of TECs or loss of the brush border and thickening of the tubular basement membrane using the following scoring system: Score 0: No tubular injury; Score 1: < 10% of tubules injured; Score 2: 10–25% of tubules injured; Score 3: 25–50% of tubules injured; Score 4: 50–74% of tubules injured; Score 5: > 75% of tubules injured.

### Immunohistochemistry staining

Three μm sections of PFA-fixed, paraffin-embedded kidney samples were deparaffinized with xylene and then rehydrated. Antigen retrieval was performed in Citrate Buffer pH 6.0 (Zytomed Systems, Bargteheide, Germany) by 10 min microwave treatment. Endogenous peroxidase activity was quenched with 3% hydrogen peroxidase at room temperature for 10 min. Slides were blocked with Blocking/Prot Block Dako (Dako, Glostrup, Denmark) for 20 min. Afterwards, the sections were incubated overnight at 4 °C with one of the antibodies listed in Supplementary Table 1. Following washing steps, slides were developed with Simple Stain MAX PO Universal Immuno-Peroxidase Polymer Histofine MaxPo anti-rabbit or anti-rat (Nichirei Biosciences, Tokyo, Japan) and visualized using diaminobenzidine substrate (Vector Laboratories, Newark, CA). For α-SMA staining, the kidney sections were incubated with a mouse monoclonal anti-α-SMA-Alkaline Phosphatase (AP) conjugated antibody (Sigma-Aldrich, St. Louis, MO; see Supplementary Table 1 for antibody details) overnight at 4 °C, and then stained with Permanent AP Red Kit (Zytomed Systems). For CD45 and FXII staining, the kidney sections were incubated with a rabbit polyclonal anti-CD45 antibody (Abcam, Cambridge, UK) or a rabbit polyclonal anti-FXII antibody (Fine Test, Wuhan, China; see Supplementary Table 1 for CD45 and FXII antibody details) overnight at 4 °C and antigen detection was performed using a ZytoChem Plus AP Polymer Kit (Zytomed Systems). The slides were counterstained with Mayer’s hematoxylin. Images were taken using the Olympus BX60 light microscope. The staining intensity or the number of positive cells were assessed in 5 images per mouse in all mice using the plugin of ImageJ/Fiji after correction for background staining [25]. The quantitative analysis of stainings was performed in a blinded fashion.

### Kidney injury molecule-1 (KIM-1) ELISA

KIM-1 levels in mouse plasma were measured using commercially available ELISA Mouse KIM-1 ELISA Kit (MSE Supplies, Tucson, AZ; cat. no.: SKU: E-EL-M3039) according to manufacturer’s instructions.

### Protein isolation and western blotting

Proteins were isolated in a lysis buffer containing 50 mM Tris, pH 7.4, 150 mM NaCl, 1 mM EDTA, 1% Triton-X-100, 1% sodium deoxycholate and 0.1% SDS, 1 mM Na_3_VO_4_, 1 mM phenylmethylsulfonyl fluoride (PMSF), and 1 μg/ml Complete Protease Inhibitor Cocktail (Roche Applied Science, Penzberg, Germany), separated on a 10% SDS polyacrylamide gel and transferred to a PVDF membrane (Carl Roth). The membrane was blocked for 1 h at room temperature with 1% non-fat milk and then incubated overnight at 4 °C with one of the antibodies listed in Supplementary Table 2. Thereafter, the membrane was probed with peroxidase-labeled secondary antibodies (all from Dako). Proteins were detected using either Amersham ECL Select Western Blotting Detection Reagent (GE Healthcare, Chicago, IL) or Pierce ECL Western Blotting Substrate (Thermo Fisher Scientific, Waltham, MA). The images were acquired using a ChemiDoc Imaging System (Bio-Rad Laboratories, Hercules, CA). The membranes were stripped and reprobed with a mouse anti-β-actin antibody (Sigma Aldrich, St. Louis, MO; see Supplementary Table 2 for antibody details) to control for loading. All quantitative measurements were conducted by investigators blinded to sample identity.

### RNA isolation and qPCR

RNA was isolated using the RNeasy Kits from Qiagen (Hilden, Germany) according to manufacturer’s instructions. cDNA was generated using qSCRIPT (Quantabio, Beverly, MA) according to manufacturer’s instructions. Quantitative real time PCR (qPCR) was performed with Blue S'Green qPCR Kit (Biozym Scientific, Hessisch Oldendorf, Germany). Primer sequences are provided in Supplementary Table 3. *Actb* or *Hprt* served as reference genes. PCR conditions were 95 °C for 10 min, followed by 40 cycles of 95 °C for 20 s, 58 °C for 30 s, and 73 °C for 30 s. All changes in target gene mRNA levels are presented as delta CT (ΔCT), which was calculated by subtracting the CT value of the target gene from the CT value of the reference gene.

### Microarray

Purified total RNA was amplified and Cy3-labeled using the LIRAK kit (Agilent Technologies, Santa Clara, CA) following the kit instructions. Per reaction, 200 ng of total RNA was used. The Cy3-labeled amplified RNA was hybridized overnight to 8 × 60 K 60-mer oligonucleotide spotted microarray slides (Agilent Technologies, design ID 074809). Hybridization and subsequent washing and drying of the slides were performed following the Agilent hybridization protocol. The dried slides were scanned at 2 µm/pixel resolution using the InnoScan is900 (Innopsys France, Carbonne, France). Image analysis was performed with Mapix 6.5.0 software, and calculated values for all spots were saved as GenePix results files. Stored data were evaluated using R software [26] and the limma package [27] from Bioconductor [28]. Mean spot signals were background subtracted and probes with negative averages in treatment groups were excluded. Quantile normalization and log2 transformation with an offset of 1 were performed and differential expression in treatment groups was assessed by Welch’s t-test (*p*-value ≤ 0.05). From different probes addressing the same NCBI gene ID, the probe showing the lowest p-value was used in subsequent analyses. Downstream overrepresentation analysis (ORA) and gene set enrichment analysis (GSEA) were performed with the clusterProfiler package [29], using a p-value threshold of 0.05 and controlling the false discovery rate (FDR) with the Benjamini–Hochberg procedure. For GSEA, genes were sorted in descending order based on fold change. Plots were created using R packages ggplot2, dplyr, circlize [30–32]. Heatmaps of z-score transformed data were created with the pheatmap package [33].The UUO kidney tissue mouse dataset GSE96101 was downloaded from the Gene Expression Omnibus (GEO) accession viewer, with preprocessing already performed by the Agilent Feature Extraction Software (A. 7.0.1). Preprocessing included background subtraction and LOWESS (Locally Weighted Scatterplot Smoothing) normalization. Differential expression analysis of complement and kallikrein-kinin related genes was conducted using Welch's t-test comparing UUO and sham group at day 3 (*p*-value ≤ 0.05). The dataset was z-score transformed prior to reporting significant expression values in a heatmap.

### Cell culture and cell stimulation

Human Embryonic Kidney (HEK) 293 cells were purchased from American Type Culture Collection (Manassas, VA) and cultured in Dulbecco´s Modified Eagle Medium (DMEM; Invitrogen Life Technologies, Carlsbad, CA) containing 10% heat-inactivated fetal calf serum (FCS; Hyclone, Cramlington, UK) and 1% Penicillin/Streptomycin (Invitrogen Life Technologies). HEK293 were cultured up to passage 23. Cryopreserved Human Renal Proximal Tubular Epithelial Cells (HRPTEpC) cells were purchased from Creative Bioarray (Shirley, NY) and cultured according to the provider’s protocol. All cells were maintained in humidified atmosphere of 5% CO_2_ at 37 °C. Prior to stimulation, the cells were seeded into a 12-well plate (2 × 10^5^ cells/well), growth-arrested in serum-free DMEM for 24 h, and then stimulated for indicated time points with 6 µg/ml FXII, 6 µg/ml FXIIa (both from CoaChrom Diagnostica, Maria Enzersdorf, Austria), or 6 µg/ml catalytically dead (CD) FXII (kindly provided by Dr. Panousis, Research and Development, CSL Limited, Bio21 Molecular Science and Biotechnology Institute, Parkville, Victoria, Australia) in the absence or presence of 10 µg/ml anti-FXIIa antibody (3F7) or 10 µg/ml IgG control (both provided by CSL Innovation). In some experiments, cells were treated with 30 µM PD98059 (MEK1/MEK2 inhibitor) or 0.5 µM LY294002 (PI3K inhibitor; both from Merck Millipore, Darmstadt, Deutschland) 1 h prior to stimulation with FXII/FXIIa. Cells were collected in 5 × loading buffer (10% SDS, 20% Glycerin, 0.05% Bromphenol blue, 0.2 M TRIS, 10% 2-Mercaptoethanol, pH 6.8) and subjected to western blotting.

### FXII/FXIIa amidolytic activity

HEK293T cells were seeded into a 96 well plate (2 × 10^4^ cells/well). Next day, they were washed with PBS and incubated 1 h at 37 °C with 6 µg/ml FXII or 6 µg/ml FXIIa (both from CoaChrom Diagnostica) in buffer containing 135 mM NaCl, 2.7 mM KCl, 11.9 mM NaHCO_3_, 0.36 mM NaH_2_PO_4_, 14.7 HEPES, 50 µM ZnCl_2_, 1 mM MgCl_2_, 2 mM CaCl_2_, 3.5 mg/ml bovine serum albumin (BSA) and 3.5 mg/ml glucose (pH 7.4). Afterwards, the cells were extensively washed with PBS and the FXIIa amidolytic activity was determined using chromogenic substrate S2302 (Chromogenix, Molndal, Sweden) according to the manufacturer’s instructions. The hydrolysis of S2302 by FXIIa was measured spectrophotometrically at 405 nm every 30 s for 60 min at 37 °C using in a SpectraMax 190 (Molecular Devices, Biberach, Germany).

### Immunocytochemistry

HEK293 cells were seeded into a 12-well plate with cover slips (2 × 10^5^ cells/well), growth-arrested in serum-free DMEM for 24 h, and then stimulated for 24 h with either 6 µg/ml FXII or 6 µg/ml FXIIa (both from CoaChrom Diagnostica). Afterwards, the cells were washed with TBS (25 mM TRIS, 137 mM NaCl, 2.7 mM KCl, pH 7.4), fixed with 4% PFA for 20 min, permeabilized with 0.1% Triton X-100 (T) for 1 h, and blocked with 1% BSA in TBS for 1 h. Subsequently, the cells were incubated overnight at 4 °C with a rabbit anti-p21 antibody (Cell Signaling, Danvers, MA; cat. no.: 2947; 1:300 in 1% BSA in TBS-T). After washing 3 × with TBS, the cells were incubated with an AlexaFluor 488-labeled anti-rabbit secondary antibody (1:300 in 1% BSA in TBS-T; Invitrogen) for 2 h at room temperature. Finally, the slides were mounted with DAPI mounting medium (Vectashield, Vector Labs, Burlingame, CA). Images were acquired on an inverted confocal laser-scanning microscope (Leica SP8, Leica Microsystems, Germany), controlled by LASX software (version 3.5.7). The quantitative analysis of staining was performed in a blinded fashion.

### Human renal sections

For the histological analysis residual renal human sections from patients suffering from hydronephrosis (*n* = 2) and normal renal tissue samples from patients who underwent tumor nephrectomy (*n* = 6) were used. Tissue sections were used after diagnostics were completed. Immunohistochemical analysis of FXII was performed as outlined above.

### Statistics 

Prior to performing parametric tests, the assumptions of normality and homogeneity of variances were assessed. Normality of each dataset was evaluated using the Shapiro–Wilk test, and homogeneity of variances across groups was assessed using the Brown-Forsythe test. For comparisons between two groups, unpaired t-tests were applied. For comparisons among three or more groups, one-way ANOVA was performed, followed by Tukey’s post hoc multiple comparisons test where appropriate. When variances were non-homogeneous, Welch's t-test (two groups) or Brown-Forsythe ANOVA followed by Dunnett’s T3 multiple comparisons test (three or more groups) were used. For non-parametric data, Mann–Whitney U test (two groups) or Kruskal–Wallis test followed by Dunn’s multiple comparisons test (three or more groups) were applied. All statistical analyses were conducted using GraphPad Prism 9 or R version 4.3.2, and a p-value < 0.05 was considered statistically significant.

## Results

**Fig. 1:**
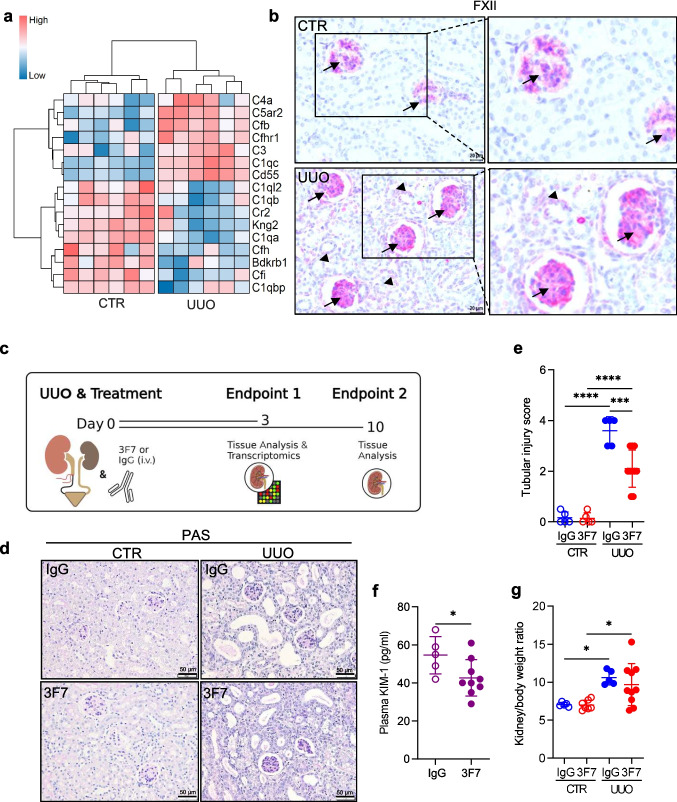
FXIIa inhibition reduces UUO-induced kidney injury. (**a**) Heatmap of genes associated with the contact phase system at day 3 post UUO (GSE96101). Expression values are z-score transformed. (**b**) Immunostaining for FXII (red color) in the glomerular mesangium (arrow) and in tubular epithelial cells (arrowhead) from ligated (UUO) and contralateral control (CTR) mouse kidneys 10 days following ligation. Scale bar 20 µm. (**c**) Schematic representation of the experimental set-up. (**d**) The extent of kidney injury demonstrated by Periodic Acid-Schiff (PAS) staining in representative kidney sections of UUO and CTR kidneys of mice treated either with IgG or 3F7. The animals were sacrificed at day 10 post ligation. Scale bar 50 µm. (**e**) Tubular injury score of PAS-stained sections. (**f**) Kidney injury molecule-1 (KIM-1) levels in plasma of mice treated either with 3F7 or IgG. The plasma samples were collected at day 10 post ligation. (**g**) Kidney/body weight ratio of mice treated either with IgG or 3F7 at day 10 post injury. *n* = 5–10/group. ****p < 0.0001; ***p < 0.001; *p < 0.05

To investigate the potential benefit of FXIIa inhibition in renal fibrosis, we first analyzed a publicly available mouse UUO microarray dataset (GSE96101, [34]) and found that the expression of various downstream mediators of FXII activation was deregulated following kidney obstruction (Fig. 1a). Specifically, several complement-related genes, including *C4a, C5ar2, Cfb, Cfhr1, C3, C1qc, Cd55, C1ql2, C1qb, Cr2, C1qa, Cfh, Cfi*, and *C1qbp*, were differentially expressed in response to kidney injury, indicating a complex regulation of the complement system in the UUO model. In parallel, *Kng2* and *Bdkrb1*, key components of the kallikrein-kinin system, were downregulated, suggesting altered bradykinin signaling in the fibrotic kidney environment. In addition, our own experimental data revealed accumulation of FXII mainly in the glomerular mesangium in control (CTR) and obstructed (UUO) kidneys on day 10 post ligation (Fig. 1b, arrows). In UUO kidneys, FXII was also detected in TECs (Fig. 1b, arrowheads). Based on these findings and previously published data showing direct contribution of FXII to the pathogenesis of DKD [15], we decided to evaluate the efficacy of 3F7 antibody in the UUO mouse model (Fig. 1c). 3F7 is the recombinant fully human antibody, which preferentially binds to FXIIa, as compared to FXII and inhibits its protease activity at the IC50 of 13 nM [24]. Ten days of ligation resulted in substantial tubulointerstitial fibrosis, which was markedly reduced upon 3F7 administration, as shown by PAS staining (Fig. 1d), and the tubular injury score (Fig. 1e). This finding was substantiated by reduced levels of KIM-1 in plasma of 3F7-treated animals as compared to mice receiving IgG (Fig. 1f). Notably, in the UUO model, one kidney is obstructed while the contralateral kidney serves as an internal control. Consequently, plasma measurements reflect systemic responses and do not permit discrimination between the control and UUO kidneys within the same animal. Therefore, any changes observed in plasma following administration of the 3F7 antibody are compared to those in IgG-treated animals and are interpreted as systemic effects resulting from FXIIa inhibition. The kidney to body weight ratio was elevated in UUO mice as compared to the control animals, however, 3F7 application had no impact on this parameter (Fig. 1g). Target engagement was demonstrated by increased levels of FXII in plasma of mice treated with 3F7 as compared to IgG (Supplementary Fig. 1a, b).

**Fig. 2:**
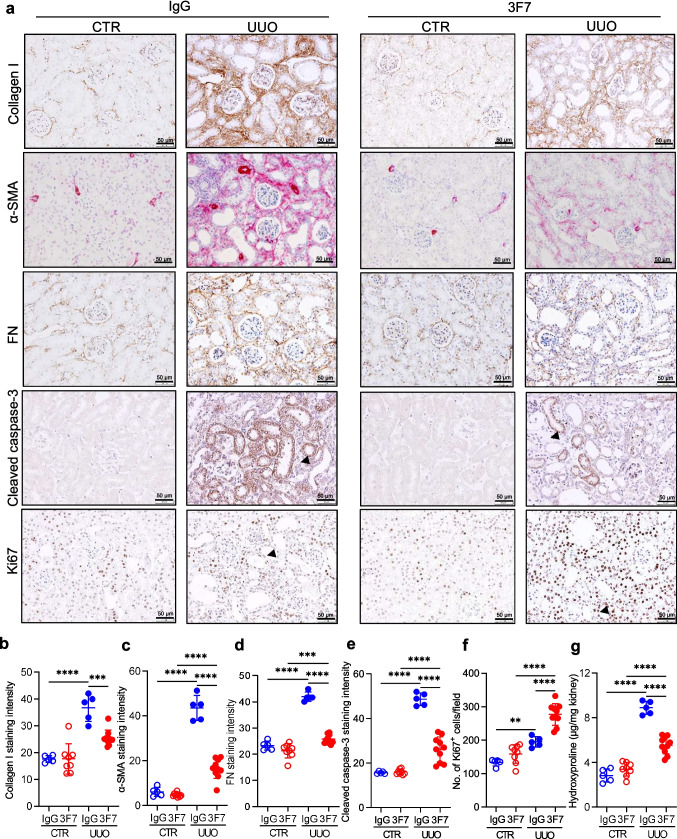
Inhibition of FXIIa reduces levels of extracellular matrix proteins and increases the number of proliferating TECs in the UUO kidney. (**a**) Collagen I, α-smooth muscle actin (α-SMA), fibronectin (FN), cleaved caspae-3, and Ki67 staining in representative kidney sections of UUO and CTR kidneys of mice treated either with IgG or 3F7. Scale bar 50 µm. (**b-f**) Collagen I (b), α-SMA (c), FN (d), cleaved caspae-3 (e), and Ki67 (f) staining quantification. (**g**) Hydroxyproline levels in UUO and CTR kidney of animals treated either with IgG or 3F7. All samples were collected at day 10 post ligation. *n* = 5–10/group. ****p < 0.0001; ***p < 0.001; *p < 0.05

To further characterize the extent of fibrosis in UUO mice treated either with 3F7 or IgG control, stainings for collagen I, fibronectin (FN) and α-smooth muscle actin (α-SMA, a marker of myofibroblasts) on kidney tissue sections were performed. Ten days of obstruction markedly elevated levels of collagen I, FN, and α-SMA in UUO kidneys (Fig. 2a, top left panels). The accumulation of collagen I, FN, and α-SMA was lower in 3F7 UUO kidneys as compared to IgG UUO kidneys (Fig. 2a, top right panels). Quantification of collagen I, FN, and α-SMA staining intensity (Fig. 2b-d) and measurement of hydroxyproline levels in kidney homogenates (Fig. 2g) confirmed these results. The attenuated fibrotic response in UUO 3F7 kidneys as opposed to UUO IgG kidneys was also observed by western blotting (Supplementary Fig. 2a-c).

Since tubulointerstitial macrophage infiltration and TEC loss correlate with the degree of inflammation and fibrosis in the UUO model [4], we also assessed the mRNA expression of inflammatory mediators (*Il1b, Il6, Tnfa, S100a8*, and *S100a9*; all selected based on their involvement in the progression of kidney fibrosis following obstruction [35, 36]), abundance of F4/80^+^ macrophages, and cell proliferation (staining for Ki67) and apoptosis (staining for cleaved-caspase 3) in CTR and UUO kidneys. Although the expression of *Il1b, Tnfa,* and *S100a9* was elevated in UUO kidneys, application of 3F7 did not have any impact on their levels (Supplementary Fig. 2d, f, and h). No significant differences in *Il6* and *S100a8* mRNA levels were observed among the experimental groups (Supplementary Fig. 2e, g). UUO kidney sections displayed strong F4/80 staining suggesting a high infiltration rate of macrophages, which was not affected by 3F7 administration (Supplementary Fig. 2i, j). While TEC apoptosis was markedly elevated in UUO kidneys and attenuated following 3F7 application (Fig. 2a, e), cell proliferation, which was increased in obstructed kidneys, was further elevated upon treatment of animals with 3F7 (Fig. 2a, f).

**Fig. 3:**
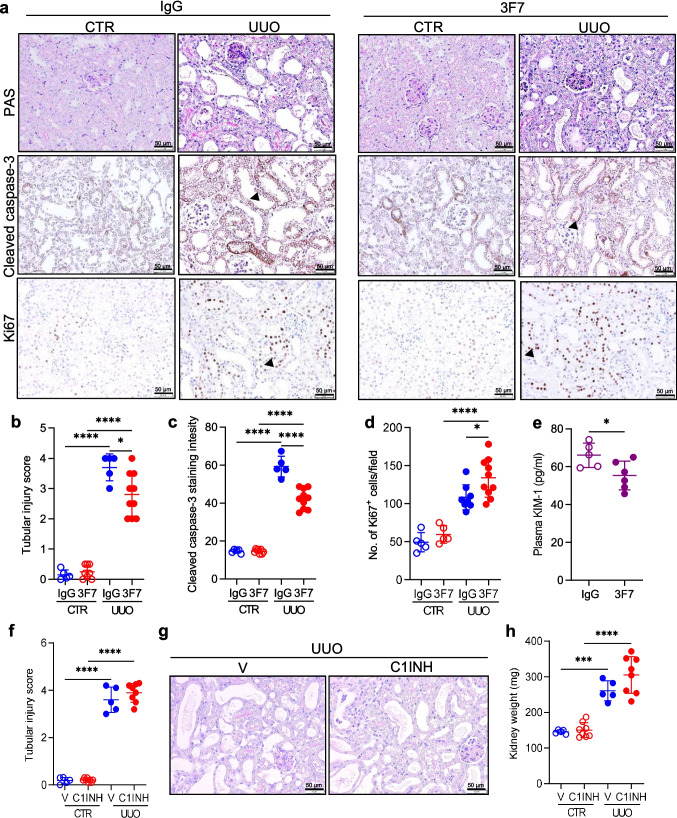
Blockage of FXIIa reduces apoptosis and promotes proliferation of TECs during the acute phase of kidney obstruction. (**a**) Periodic Acid-Schiff (PAS), cleaved caspase-3, and Ki67 staining in representative kidney sections of UUO and CTR kidneys of mice treated either with IgG or 3F7. Scale bar 50 µm. (**b**) Tubular injury score of PAS-stained sections. (**c**, **d**) Cleaved caspase-3 (c) and Ki67 (d) staining quantification. (**e**) Kidney injury molecule-1 (KIM-1) levels in plasma of mice treated with either with 3F7 or IgG. (**f**) Kidney weight of mice treated either with vehicle (V; 0.9% NaCl) or C1 esterase inhibitor (C1INH). (**g**) The extent of kidney injury as demonstrated by PAS staining in representative kidney sections of UUO and CTR kidneys of mice treated either with V or C1INH. Scale bar 50 µm. (**h**) Tubular injury score of PAS-stained sections. All samples were collected at day 3 post ligation. *n* = 5–10/group. ****p < 0.0001; ***p < 0.001; **p < 0.01; *p < 0.05

To gain insights into the molecular mechanisms underlying the protective effects of 3F7 in the UUO mouse model, kidney tissue injury was also evaluated at day 3 post obstruction, and thus at the time before fibrosis manifests. As shown in Fig. 3a (top left panels) and b, UUO mice displayed marked kidney tubular injury as compared to controls. These pathological alterations were diminished upon 3F7 administration (Fig. 3a (top right panels), b). Further analysis revealed increased intensity of cleaved-caspase 3 staining (Fig. 3a, c) and no change in the number of Ki67^+^ cells (Fig. 3a, d) in UUO kidneys as opposed to controls. Application of 3F7 markedly lowered apoptosis (Fig. 3a, c) and increased proliferation (Fig. 3a, d) in UUO kidneys when compared to UUO IgG. KIM-1 levels were also decreased in plasma of animals treated with 3F7 as compared to IgG (Fig. 3e). However, 3F7 administration neither affected the accumulation of inflammatory cells (CD45⁺) in general, and F4/80⁺ macrophages in particular (Supplementary Fig. 3a–d), nor did it alter the mRNA expression of *Il1b, Il6, Tnfa, Ccl2*, or *Cxcl1* in UUO kidneys relative to UUO IgG (Supplementary Fig. 4a–e). Nonetheless, *S100a8* and *S100a9* mRNA levels in obstructed kidneys were reduced following 3F7 application, supporting the protective effect of FXIIa inhibition on TEC (Supplementary Fig. 4f, g) [35]. Previous studies have reported that exposure of TECs to S100A8/A9 promotes cell cycle arrest and even cell death, whereas loss of S100A8/A9 protects TEC from UUO-induced apoptosis and epithelial–mesenchymal transition [35]. Strikingly, neither kidney weight nor the tubular injury score were altered in UUO kidneys by the application of the C1 esterase inhibitor (C1INH; Fig. 3f-h), the main physiological inhibitor of FXIIa and the complement and kallikrein-kinin system [37]. Likewise, no difference in the expression of the selected inflammatory mediators in UUO kidneys as compared to UUO plus vehicle (V) was observed (Supplementary Fig. 5a-c). These results suggest that selective blockade of FXII/FXIIa is favorable when compared to simultaneous inhibition of the contact phase, complement, and kallikrein–kinin systems, and that the profibrotic effects of FXII/FXIIa are largely independent of its kallikrein–kinin and complement system–activating properties.

**Fig. 4:**
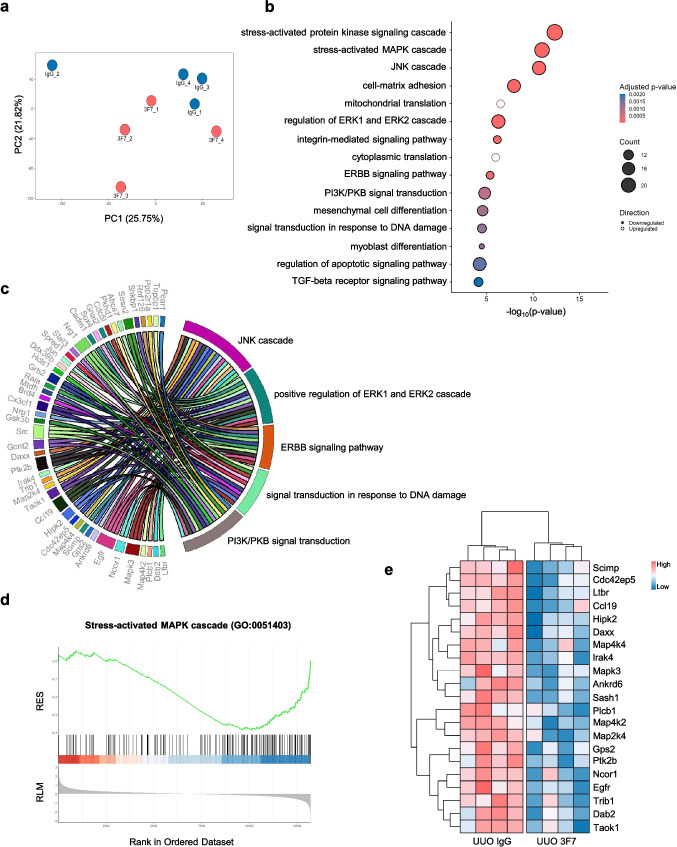
Microarray data of UUO kidney samples confirm the efficacy of 3F7 in reducing profibrotic and stress-mediated signaling pathways. (**a**) Principal component analysis (PCA) of sample gene expression. (**b**) Overrepresentation analysis (ORA) pathways of significantly up- and downregulated genes highlight the reduction of profibrotic and stress-mediated signaling. (**c**) Chord plot illustrating the interconnectivity of downregulated genes across pathways, including the JNK cascade, positive regulation of ERK1 and ERK2 cascade, ERBB signaling pathway, signal transduction in response to DNA damage, and PI3K/PKB signal transduction. (**d**) Stress-activated MAPK signaling cascade related gene expression is downregulated upon 3F7 treatment in gene set enrichment analysis (GSEA). The x-axis displays gene ranks based on log2 fold change. RLM = Ranked list metric, displaying gene expression fold change. RES = Running enrichment score, indicated by the green line. (**e**) Z-score transformed expression values of significantly downregulated genes in the GO pathway, stress-activated MAPK signaling cascade (GO:0051403). Kidney tissue was collected at day 3 post ligation. *n* = 4/group

Gene expression analysis of UUO kidney tissue homogenates (KH) was conducted on day 3 post ligation to gain insights into the signaling pathways affected by 3F7 treatment compared to the IgG control. KH samples are heterogeneous tissue samples that can be used to identify individual genes, revealing treatment-associated alterations in expression, pointing to processes that are either directly or indirectly changed by 3F7. A comparison of the entire gene expression profiles by principal component analysis (PCA) revealed clustering of three biological replicates for each treatment group, with one deviant sample identified in both antibody-treated groups (Fig. 4a). All biological replicates were included in the downstream analysis. Gene Ontology (GO) pathway enrichment showed a number of FXII-associated pathways significantly altered following 3F7 treatment (Fig. 4b). These included stress-activated protein kinase signaling/MAPK cascade, JNK cascade, regulation of ERK1 and ERK2 cascade, ErbB signaling pathway, PI3K/protein kinase B (Akt) signal transduction, or signal transduction in response to DNA damage. The interconnectivity among these pathways is illustrated in a chord plot provided in Fig. 4c. GSEA of GO pathways highlighted significant changes in the stress-activated MAPK signaling cascade (Fig. 4d), ERK1 and ERK2 signaling cascade (Supplementary Fig. 6a) and DNA damage response (Supplementary Fig. 6b). Genes downregulated upon 3F7 treatment in the GO pathway stress-activated MAPK signaling cascade are shown in Fig. 4e.

**Fig. 5:**
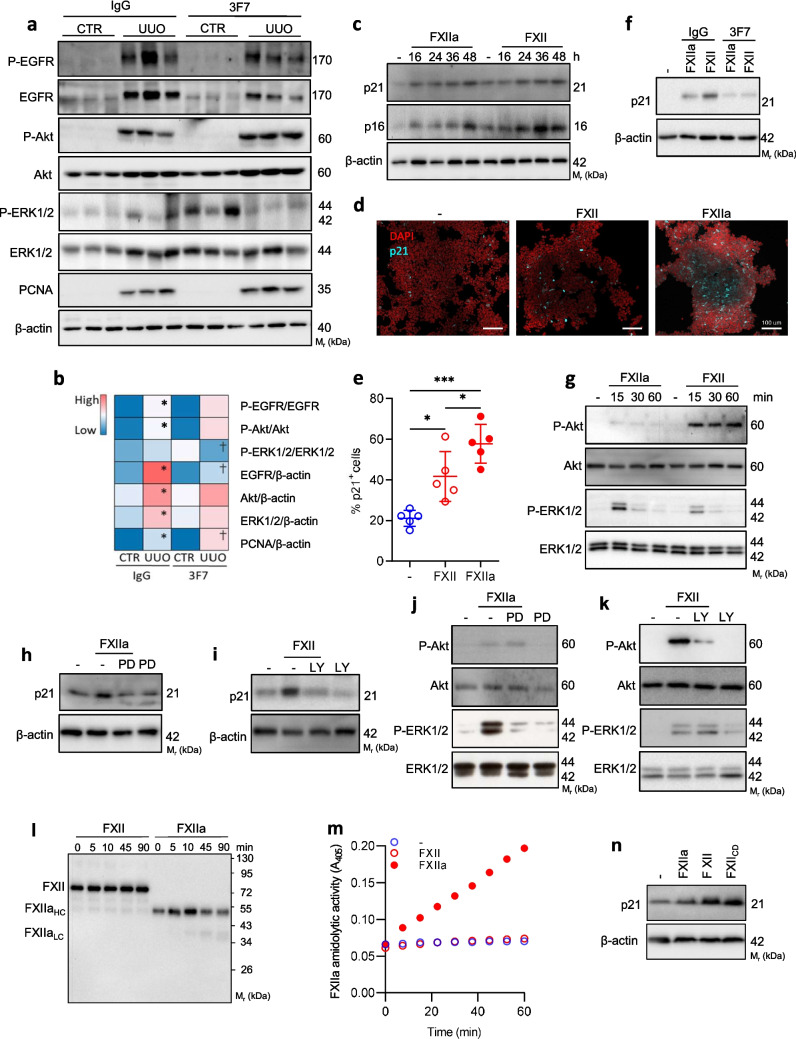
FXIIa and FXII trigger p21 expression in renal epithelial cells. (**a**) Phosphorylated (P) and total protein levels of EGFR, Akt, ERK1/2, and PCNA in UUO and CTR kidneys of animals injected either with IgG or 3F7. The animals were sacrificed at day 3 post ligation. Representative western blots are shown. (**b**) Densitometric evaluation of western blots shown in (a) visualized in a heatmap. *n* = 5/group. *IgG CTR vs IgG UUO (*p* < 0.05), †IgG UUO vs 3F7 UUO (*p* < 0.05). (**c**) Expression of p21 and p16 in HEK293 cells stimulated with FXIIa or FXII. β-actin was used as a loading control. Data are representative of three independent experiments. (**d**) p21 staining in FXIIa or FXII-stimulated HEK293 cells. (**e**) Percentage of p21.^+^ HEK293 cells upon stimulation with FXIIa or FXII; calculated from five independent experiments. (**f**) Expression of p21 in HEK293 cells stimulated with FXIIa/FXII in the absence or presence of 3F7 or IgG. β-actin was used as a loading control. Data are representative of three independent experiments. (**g**) Phosphorylated and total protein levels of Akt and ERK1/2 in HEK293 cells treated for the indicated time points with FXIIa or FXII. Phosphoproteins were detected with phospho-specific antibodies. Equal loading was confirmed with pan-specific antibodies. Data are representative of three independent experiments. (**h**, **i**) Expression of p21 in HEK293 cells stimulated with FXIIa or FXII in the absence or presence of MEK inhibitor PD98059 (PD) or PI3K/Akt inhibitor LY294002 (LY). β-actin was used as a loading control. Data are representative of three independent experiments. (**j**, **k**) Western blots showing P-Akt and P-ERK1/2 in HEK293 cells stimulated with FXIIa or FXII in the absence or presence of PD or LY. Phosphoproteins were detected with phospho-specific antibodies. Equal loading was confirmed with pan-specific antibodies. Data are representative of three independent experiments. (**l**) Stability of FXII during 90 min incubation at the HEK293T cell monolayer. Data are representative of three independent experiments. FXII_HC_, FXII heavy chain; FXII_LC_, FXII light chain. (**m**) FXIIa amidolytic activity measured at the HEK293T cell monolayer. The hydrolysis of S2302 by FXIIa was measured spectrophotometrically at 405 nm (A_405_). One representative experiment out of three is shown. (**n**) Expression of p21 in HEK293T cells stimulated with FXII, FXIIa, or catalytically dead FXII (FXII_CD_). β-actin was used as a loading control. Data are representative of three independent experiments. ***p < 0.001; *p < 0.05

MAPK, ERK1 (also known as MAPK3)/ERK2, PI3K and ErbB/EGFR signaling pathways as well as signal transduction in response to DNA damage have been reported as essential for the transition from acute to chronic kidney injury [38] and, in addition, they have been linked to cellular activities of FXII/FXIIa across various pathologies [20, 21]. Based on these assumptions, we decided to validate the expression of ERK1/ERK2, Akt and EGFR in UUO and CTR kidneys on the protein level at day 3 post ligation. As depicted in Fig. 5a the levels of EGFR, Akt and ERK1 were markedly elevated in UUO kidneys and compared to CTR. While 3F7 administration reduced EGFR levels, it did not have any impact on Akt and ERK1 expression in diseased organs. Furthermore, there was no change in the activity of EGFR and Akt but a reduction in the phosphorylation of ERK1 in UUO 3F7 kidneys as compared to UUO IgG kidneys (Fig. 5a, b and Supplementary Fig. 7a-f). Western blot analysis confirmed increased expression of PCNA, a marker of cell proliferation, in UUO 3F7 kidneys as opposed to UUO IgG control (Fig. 5a, b and Supplementary Fig. 7 g). Of note, the low abundance of ERK2 and its comigration with ERK1 likely precluded its detection by western blotting in KH. Moreover, we cannot exclude the possibility that ERK2 is not involved in the biological processes explored.

Next, we investigated the functional relevance of FXII in kidney injury. Based on the analysis of our transcriptome data and previously published results demonstrating induction of cellular senescence upon exposure of renal tubular cells to FXIIa [15], we decided to evaluate the capacity of FXIIa and FXII to induce senescence of human-derived renal epithelial cells (HEK293). Indeed, exposure of HEK293 to FXIIa and FXII induced expression of p21 and p16 (both known as markers of senescence) in a time-dependent manner (Fig. 5c). These results were further corroborated by the increased number of p21^+^ cells (Fig. 5d, e). FXII/FXIIa-triggered p21 expression was diminished by the application of 3F7 (Fig. 5f). While stimulation of the cells with FXIIa induced marked phosphorylation of ERK1/2 that reached a maximum within 10–15 min, the pronounced and sustained phosphorylation of Akt (remaining above baseline up to 60 min) was only observed upon exposure of cells to FXII (Fig. 5g). These results imply that FXIIa and FXII demonstrate different kinetics and magnitude of activation of intracellular signaling mediators, with FXII favoring Akt over ERK1/2 and, vice versa, FXIIa favoring ERK1/2 over Akt. Accordingly, blockage of ERK1/2 with MEK inhibitor PD98059 and Akt with PI3K/Akt inhibitor LY294002 abolished FXIIa- and FXII-induced p21 expression, respectively (Fig. 5h, i). Furthermore, PD98059 reduced FXIIa-triggered ERK1/2 phosphorylation, however, it did not affect Akt activation (Fig. 5j). Conversely, LY294002 markedly diminished Akt phosphorylation and did not impact ERK1/2 activation following stimulation of the cells with FXII (Fig. 5k). To ensure that FXII-induced Akt phosphorylation is induced by FXII zymogen and not FXIIa protease generated during FXII incubation with the cells, the stability of FXII zymogen on a HEK293 monolayer was evaluated by western blotting and utilizing a chromogenic substrate. As depicted in Fig. 5l and m, FXII zymogen (80 kDa protein) levels remained stable even after 90 min of incubation and it did not develop amidolytic activity over 60 min of exposure to the cells. Furthermore, the induction of p21 expression was also observed upon stimulation of the cells with catalytically dead FXII (FXIICD; Fig. 5n) ultimately indicating that FXII catalytic activity is not required for its cellular functions.

**Fig. 6:**
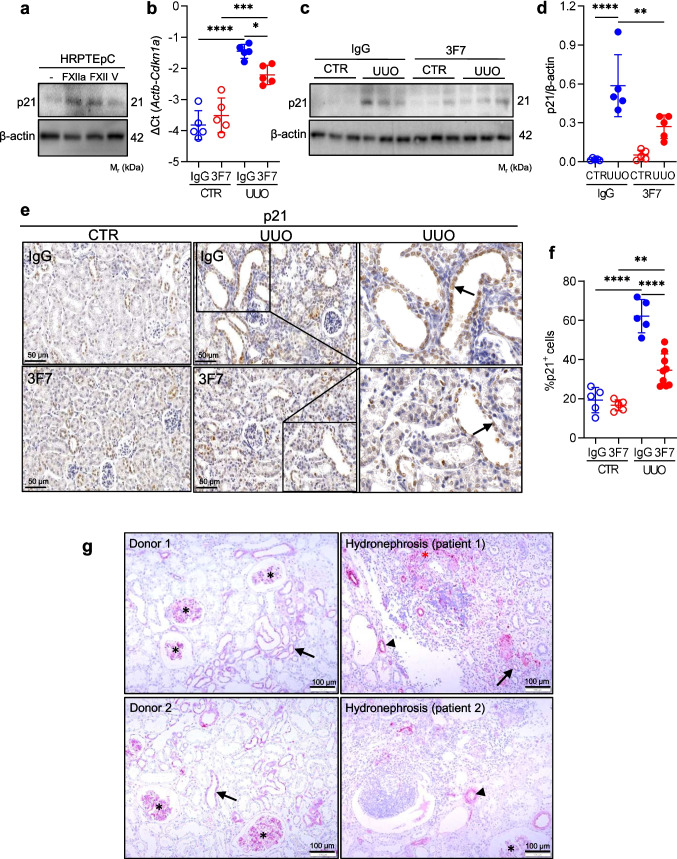
3F7 reduces the number of p21.^+^ cells in UUO kidneys. (**a**) Western blots showing expression of p21 in HRPTEpC cells stimulated with FXII, FXIIa, or vehicle (V). β-actin was used as a loading control. Data are representative of three independent experiments. (**b**) mRNA expression of *Cdkn1a* in UUO and CTR kidneys of mice treated either with IgG or 3F7 at day 10 post ligation. The qPCR data are presented as a ΔCt using *Actb* (β-actin) as a reference gene. (**c**) Western blot showing p21 in UUO and CTR kidneys of animals injected either with IgG or 3F7 at day 10 post obstruction. β-actin was used as a loading control. Representative western blots are shown. (**d**) Densitometric evaluation of western blots shown in (c). (**e**) p21 staining in representative UUO and CTR kidney sections of mice treated either with IgG or 3F7 at day 10 post ligation. Scale bar 50 µm. (**f**) p21 staining quantification. (**g**) FXII immunostaining in human renal sections from patients suffering from hydronephrosis (*n* = 2) and normal kidney tissue (donor; *n* = 6). An asterisk depicts the localization of FXII in the glomerular mesangium, an arrow indicates tubular epithelial cells, and an arrowhead points to peritubular vessels. The red asterisk shows a fibrotic lesion in the renal interstitium. Scale bar 100 µm. *n* = 5–10/group ****p < 0.0001, ***p < 0.001; *p < 0.05

The induction of p21 protein expression was also observed upon exposure of primary human renal proximal epithelial cells to either FXIIa or FXII (Fig. 6a), demonstrating relevance of our findings in more physiological conditions. Finally, elevated p21 levels were observed in UUO kidneys, as assessed by qPCR (Fig. 6b), western blotting (Fig. 6c, d), and immunohistochemistry (Fig. 6e, f). The p21 signal was markedly diminished in UUO 3F7 kidneys as compared to UUO IgG kidneys (Fig. 6b-f). Immunostaining for FXII was also performed in normal renal tissue sections from patients who underwent tumor nephrectomy and patients suffering from hydronephrosis. FXII was primarily localized in the glomerular mesangium, similar to its distribution in the murine kidney (Fig. 6g). Strikingly, in normal kidney tissue, a FXII-positive signal was occasionally observed in TECs, whereas in diseased kidney, it was also concentrated around peritubular vessels and fibrotic lesions in the renal interstitium (Fig. 6g).

## Discussion

Kidney fibrosis is a common manifestation of a wide variety of CKD with parenchymal tissue scarring serving as a histologic predictor of functional deterioration [39]. Furthermore, even though the deposition of the extracellular matrix (ECM) following initial injury may support the repair and regeneration, persistent and uncontrolled scar tissue formation may fully disrupt organ architecture and ultimately lead to kidney failure [39]. Given the high prevalence of CKD [1], there is a high unmet clinical need for new therapies to treat this disease [3, 5]. This also includes diabetic nephropathy [40], the primary cause of CKD in developed countries, even though several drugs, such as sodium glucose cotransporter 2 inhibitor (SGLT2i), glucagon-like peptide 1 receptor agonist (GLP-1 RA) or angiotensin II receptor blockers (ARB), were found to reduce the risk of kidney failure [40, 41]. Considering the relationships between contact phase system activation and renal fibrosis [42] as well as potential pro-fibrotic activities of FXII [15, 19–21], we hypothesized that the inhibition of FXIIa with the anti-FXIIa antibody 3F7 may demonstrate therapeutic efficacy in the mouse model of UUO.

Treatment of UUO animals with 3F7 showed therapeutic potential as evidenced by preserved tissue structure, diminished apoptosis of TECs and decreased accumulation of ECM proteins in injured kidneys. Based on our findings demonstrating no changes in the expression of the inflammatory molecules, lack of complement and kininogen/kallikrein signature in the transcriptome dataset, and no efficacy of C1INH in the acute phase of the UUO model, we concluded that the beneficial effects of 3F7 are associated with non-canonical functions of FXII/FXIIa related to its ability to activate intracellular signaling pathways and thereby to control cellular processes. Indeed, FXII/FXIIa was shown to interact with uPAR and transduce signaling following uPAR-β1 integrin-EGFR complex formation. Although data on the anti-fibrotic effects of intervening with uPAR in various organs including the kidney are conflicting [43], clear favorable results have been obtained in the studies using strategies to block EGFR [44, 45]. For example, waved-2 mice, which contain a point mutation in EGFR that reduces receptor tyrosine kinase activity by > 90%, or those with fibroblast-specific EGFR deletion have been demonstrated to develop less kidney fibrosis in the UUO model [45, 46]. Moreover, inhibition of renal fibrosis has been observed in UUO mice treated with the EGFR inhibitor gefitinib [44, 45]. Similar anti-fibrotic effects of EGFR blockade have been also observed in other murine models of kidney diseases including angiotensin II-infused mice [47].

The beneficial effects of FXIIa inhibition in the UUO model were dependent neither on CD45^+^ or F4/80 macrophage numbers, otherwise known to play a prominent role in renal fibrosis [48], nor inflammation, even though anti-inflammatory effects of 3F7 have been reported [49]. Mice treated with 3F7 exhibited markedly fewer apoptotic TECs, decreased expression of S100a8/S100a9, and a substantial increase in proliferating TECs, resulting in reduced tubular atrophy and improved renal morphology. These findings highlight the pivotal role of FXIIa in regulating cellular processes related to TEC (patho)biology. Accordingly, *S100a9*-/^−^ mice were found to be protected from UUO-induced renal fibrosis independently of leucocyte infiltration and inflammation and loss of S100A8/A9 shielded TEC from UUO-induced apoptosis and epithelial-mesenchymal transition. In vitro studies further demonstrated that exposure of TECs to S100A8/A9 can induce cell cycle arrest and even cell death [35]. Collectively, these findings strongly support our data showing the major impact of the anti-FXIIa therapy on TEC “well-being” and suggest that both direct and indirect effects mediated by the downregulation of S100A8/A9 expression may have contributed to the efficacy of the 3F7 antibody in the UUO mice.

Recent findings suggest that TECs, rather than acting as victims, represent the driving force in the progression of kidney diseases [6–8]. Following severe and recurrent injuries, TECs undergo structural and phenotypic changes, which are accompanied by altered expression/activity of profibrotic factors [6–8]. Among them, EGFR, PI3K/Akt, and ERK1/2 were found to play a prominent role [21, 50]. Strikingly, the aforementioned pathways were found to be regulated by 3F7 application in the course of kidney fibrosis development. Gene expression profiling revealed alterations in stress-activated protein kinase signaling cascades, ERK1/2 and PI3K signaling cascades, ErbB signaling pathway, regulation of apoptotic signaling pathway, and signal transduction in response to DNA damage. These findings were corroborated by in situ studies showing prominent downregulation of EGFR expression and ERK1 activity in UUO kidneys following 3F7 administration. Diminished EGFR expression and downstream intracellular signaling may thus be one of the underlying mechanisms responsible for attenuated tissue damage response to ureteral obstruction, contributing to the markedly reduced kidney fibrosis in 3F7-treated mice. The concomitant increase in proliferating TECs further suggests that FXIIa inhibition facilitates the transition from injury to repair, potentially through regulation of the EGFR- and ERK1/Akt-mediated responses. In line with this notion, Xu et al. reported that deregulation of EGFR expression and activation is crucial for the switch from acute to chronic kidney injury [38]. At present, we can only speculate about the strong increase in ERK1 activity following 3F7 administration in CTR kidneys. Other studies [15, 21] together with our unpublished data suggest that FXII/FXIIa-driven intracellular signaling is mediated through the formation of an uPAR/β1 integrin/EGFR complex. Since these receptors can also interact with other ligands [51–53], it is conceivable that the balance between FXII/FXIIa and alternative ligands influences overall ERK1 pathway activity. This observation warrants further spatiotemporal analysis of uPAR/β1 integrin/EGFR receptor–ligand interactions in health and disease.

One of the reactions of cells to stress and DNA damage is transition to a senescent phenotype. Senescence has recently been described in human and murine kidney TEC exposed to FXII [15]. Accordingly, *f12*-deficient mice were found to be protected from hyperglycemia-induced kidney injury and urinary FXII levels positively correlated with kidney dysfunction in DKD [15]. Analysis of genes regulated by 3F7 in UUO kidneys revealed a number of potential links to cellular senescence. For example, *Hipk2* has been shown to control DNA damage-provoked cell fate by inducing either proapoptotic or prosenescent posttranslational modifications of p53 [54]. Likewise, *Daxx* has been reported to regulate apoptosis and replicative senescence in response to agents triggering DNA destruction [55]. Furthermore, inflammation- or DNA damage-induced uncontrolled EGFR- and ERK1/2-mediated signaling has been found to drive cellular senescence in lung fibroblasts and human umbilical vein endothelial cells [56]. In addition, prolonged or exacerbated activation of ERK1/2 signaling has also been associated with cellular senescence in human cervical cancer cells [57] and in pancreatic adenocarcinoma cells [58]. Although no data on the role of EGFR/ERK1/2/Akt in the induction of a senescence phenotype of TECs have been reported yet, our findings strengthen the importance of temporal regulation of the expression and activities of the aforementioned molecules in the transition from adaptive to maladaptive tissue repair.

Consistent with previous reports [19, 21], we demonstrate the central role of Akt and ERK1/2 signaling in FXII/FXIIa-triggered cellular effects. The kinetics and magnitude of Akt and ERK1/2 activation differed, depending on whether renal epithelial cells were exposed to FXII or FXIIa, with FXII preferentially activating Akt and FXIIa favoring ERK1/2. While the underlying molecular mechanisms remain speculative, conformational rearrangements associated with FXII activation [59] could influence the kinetics and affinity of FXIIa binding to its receptor, thereby initiating specific signaling pathways. On the other hand, FXII/FXIIa has been reported to interact with a multiprotein receptor complex on the cell surface [21], thus FXII/FXIIa conformation could not only dictate the kinetics and binding affinity but also the overall composition of the receptor complex. Further studies dissecting the structural nature and temporal dynamics of FXII- versus FXIIa-receptor interactions are required to fully explore this phenomenon.

Finally, we demonstrated FXII-positive staining in TECs, peritubular vessels, and fibrotic lesions within renal interstitium of patients with hydronephrosis. The increased local expression of FXII and/or elevated glomerular filtration rate of plasma-derived FXII may represent potential sources of FXII in a diseased kidney. Local synthesis of FXII by non-hepatic cells has been reported [15, 60] and the transport of FXII across an impaired glomerular filtration barrier cannot be excluded, particularly since urinary FXII levels have been shown to correlate with kidney function in DKD patients [15]. Moreover, previous studies have demonstrated that FXII can bind to endothelial cells [61] as well as to collagen fibers exposed following endothelial cell injury [62]. These mechanisms may therefore account for the enhanced FXII immunoreactivity detected in the peritubular vessels of diseased kidneys. This assumption is further supported by reports describing endothelial cell damage such as apoptosis and a loss of pro-angiogenic potential in patients with CKD [63]. Whether the increased FXII immunoreactivity in the peritubular vessels contributes to the prothrombotic state reported in CKD patients or instead participates in the regulation of other cellular processes remains to be elucidated in future studies.

Our study has several limitations. It relies on a single obstructive model of kidney fibrosis that reproduces key pathological features of CKD, including tubular injury and atrophy, but has limited translational relevance given the rarity of complete ureteral obstruction in humans. The human data are correlative and based on a very small sample size (n = 2), precluding definitive interpretation. Validation in larger and more diverse CKD cohorts will be essential to establish the clinical relevance of our findings. The in vitro experiments were performed in HEK293 cells, which do not fully recapitulate the phenotype of primary TECs, therefore, further studies using primary cell or tissue culture models are required to delineate the molecular mechanisms regulating p21 expression upon exposure to FXII or FXIIa. Our findings provide an initial framework for understanding TEC alterations following FXII/FXIIa blockade. Future studies should define the FXII/FXIIa-dependent phenotypic consequences in TECs, including activation of cellular stress pathways leading to senescence or cell death.

In conclusion, our study demonstrates the efficacy of 3F7 in the UUO model and may facilitate the translation of insights gleaned from this model into clinical development. Notably, the anti-FXIIa antibody, Garadacimab, was recently approved for routine prevention of recurrent attacks of hereditary angioedema [51]. Although current therapeutic strategies such as SGLT2i, GLP-1 RA, renin-angiotensin system blockade, tolvaptan (vasopressin receptor 2 antagonist), atrasentan (endothelin-1 blocker), or finerenone (non-steroidal anti-mineralocorticoid) can decelerate CKD, none are capable of halting disease progression [3, 41]. Therefore, the development of innovative therapeutic approaches remains essential. The knowledge gained from this study is an important step forward in understanding the clinical benefits of anti-FXIIa antibody therapy and provides a rationale for evaluating across other pathological conditions.

## Supplementary Information

Below is the link to the electronic supplementary material.

Supplementary file1 (PDF 1407 KB)

## Data Availability

The datasets generated during the current study are not publicly available due to CSL ownership rights but are available from the corresponding author on reasonable request.

## Abbreviations

α-SMA: α-smooth muscle actin
ARB: Angiotensin II receptor blockers
BK: Bradykinin
BSA: Bovine serum albumin
C1INH: C1 esterase Inhibitor
CD: Catalytically dead
CKD: Chronic kidney disease
CTR: Control
DKD: Diabetic kidney disease
DMEM: Dulbecco’s modified eagle medium
ECM: Extracellular matrix
EGFR: Epidermal growth factor receptor
FCS: Fetal calf Serum
FN: Fibronectin
FXII(a): (Activated) factor XII
GEO: Gene expression omnibus
GLP-1 RA: Glucagon-like peptide 1 receptor agonist
GSEA: Gene set enrichment analysis
HEK: Human embryonic kidney
HRPTEpC: Human renal proximal tubular epithelial cells
KH: Kidney tissue homogenates
KIM-1: Kidney injury molecule-1
ORA: Overrepresentation analysis
PFA: Paraformaldehyde
PAS: Periodic acid-schiff
RES: Running enrichment score
RLM: Ranked list metric
SGLT2i: Sodium glucose cotransporter 2 inhibitor
TECs: Tubular epithelial cells
uPAR: Urokinase receptor
UUO: Unilateral ureteral obstruction

## Declarations

The study was performed in accordance with the declaration of Helsinki and the usage of human biospecimen was approved by the institutional review board (Medical Faculty, Goethe University, Frankfurt, Germany, no.: 2023–1135). The animal study was approved by the governmental ethics committee for animal welfare (Regierungspräsidium Darmstadt, Germany; no.: V 54—19 c 20/15 – FU/1063).
