# Supplementary material for: Inhibition of FXIIa attenuates kidney fibrosis in mice with unilateral ureteral obstruction
Source: Cell Mol Life Sci. 2025 Nov 29;83(1):21. doi: 10.1007/s00018-025-05988-z (PMC12775217; doi:10.1007/s00018-025-05988-z)
Supplement: Supplementary file 1 — Supplementary file1 (PDF 1407 KB) [file 18_2025_5988_MOESM1_ESM.pdf]

Supplementary material

Inhibition of FXIIa attenuates kidney fibrosis in mice with unilateral ureteral obstruction

Daniel Kalina[1], Daniel P. Potaczek[2,3], Jinyang Zeng-Brouwers[4], Mario Boehm[1], Marc W. Nolte[1], Maximilian Bielohuby[1], Ralph T. Schermuly[5,6], Liliana Schafer[4], Malgorzata Wygrecka[1,2,5,6]

[1]CSL Innovation GmbH, Marburg, Germany. [2]Center for Infection and Genomics of the Lung (CIGL), Faculty of Medicine, Justus Liebig University (JLU), Giessen, Germany. [3]Translational Inflammation Research Division & Core Facility for Single Cell Multiomics, Medical Faculty, Philipps-University Marburg, Marburg, Germany. [4]Institute of Pharmacology and Toxicology, Goethe University, Frankfurt, Germany. [5]Department of internal Medicine, Justus Liebig University of Giessen, Giessen, Germany. Member of the German Center for Lung Research. [6]Institute for Lung Health (ILH), Justus Liebig University (JLU), Giessen, Germany.

Supplementary Table 1. Antibodies used for immunohistochemistry

Antibody	Company	Catalog No.	Lot No.	Dilution
Collagen I	Abcam	ab34710	GR271609-5	1:500
α-SMA-AP	Sigma	A5691	117M4870V	1:100
Fibronectin (FN)	Abcam	ab2413	GR3454892-1	1:250
Caspase 3 (cleaved)	Cell Signaling	9662	19	1:100
Ki67	Abcam	ab16667	GR341233 21	1:200
F4/80	BIO-RAD	MCA497G	1605	1:50
FXII	Fine Test	FNab02938		1:200
p21	Cell Signaling	2947	14	1:300
CD45	Abcam	ab10558	GR323033-1	1:200

Supplementary Table 2. Antibodies used for western blotting

Antibody	Company	Catalog No.	Lot No.	Dilution
P-Akt (S473)	Cell Signalling	4060	9	1:500
t Akt	Cell Signalling	2966	5	1:1000
PCNA	Cell Signalling	2586	7	1:1000
β-actin	Sigma	A1978-100UL	0000137631	1:5000
P-p44/42 (T202/Y204)	Cell Signalling	9106	30	1:2000
p44/42	Cell Signalling	4695	14	1:1000
p21	Cell Signalling	2947	14	1:1000
EGFR	Cell Signalling	4267	24	1:1000
P-EGFR (Y1068)	Abcam	Ab40815		1:1000
FXII	Zytomed Systems	206-0056	17040653	1:1000

Supplementary Table 3. qPCR mouse primers

Gene	Accession number	Nucleotide Sequence (5'-3')
Tnf_F	NM_013693.3	CGAGTGACAAGCCTGTAGCC
Tnf_R	NM_013693.3	CTTTGAGATCCATGCCGTTG
Il1b_F	NM_008361.4	GGAAGCAGCCCTTCATCTTT
Il1b_R	NM_008361.4	TGGCAACTGTTCCTGAACTC
Il6_F	NM_031168.2	CAAAGCCAGAGTCCTTCAGAG
Il6_R	NM_031168.2	AGGAGAGCATTGGAAATTGG
Cdkn1a_F	NM_007669.5	GGAACATCTCAGGGCCGAAA
Cdkn1a_R	NM_007669.5	CTGACCCACAGCAGAAGAGG
Ccl2_F	NM_011333.3	CACTCACCTGCTGCTACTCA
Ccl2_R	NM_011333.3	GCTTGGTGACAAAAACTACAGC
Cxcl1_F	NM_008176.3	AGACCATGGCTGGGATTC
Cxcl1_R	NM_008176.3	GAGTGTGGCTATGACTTC
Hprt_F	NM_013556.2	GCTGACCTGCTGGATTAC
Hprt_R	NM_013556.2	TTGGGGCTGTACTGCTTA
Actb_F	NM_007393.5	CCGCGAGCACAGCTTCTTTG
Actb_R	NM_007393.5	GCCCACGATGGAGGGGAATAC
S100a8_F	NM_013650.2	GTCCTCAGTTTGTGCAGAATATAAA
S100a8_R	NM_013650.2	GCCAGAAGCTCTGCTACTCC
S100a9_F	NM_009114.3	AGGAAGGAAGGACACCCTGA
S100a9_R	NM_009114.3	TGTGTCCAGGTCCTCCATGA

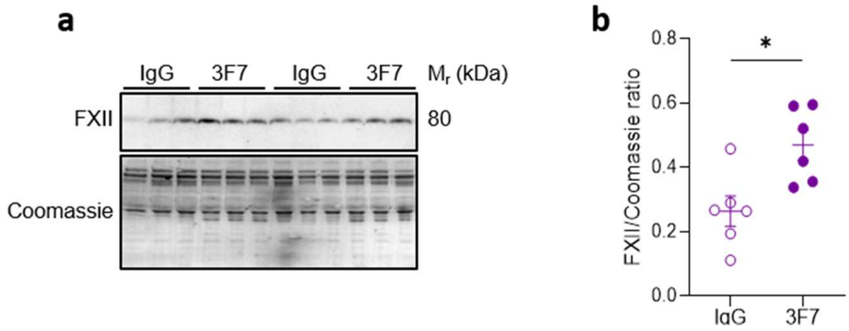

Supplementary Figure 1. Target engagement in plasma of IgG- and 3F7-trated mice subjected to UUO. a) Western blot showing FXII levels in plasma of mice either treated with IgG or 3F7 at day 10 post ligation. Mice received daily either IgG or 3F7 starting directly after ligation. Coomassie stained membrane was used to evaluate loading of the proteins on the gel. b) Quantification of western blot shown in (a). Notably, plasma levels of FXII reflect systemic changes and do not allow distinguishing between the obstructed and contralateral (control) kidneys in the UUO model. To indicate this visually we used a violet color - a combination of blue and red. n=6/group. *$p<0.05$.

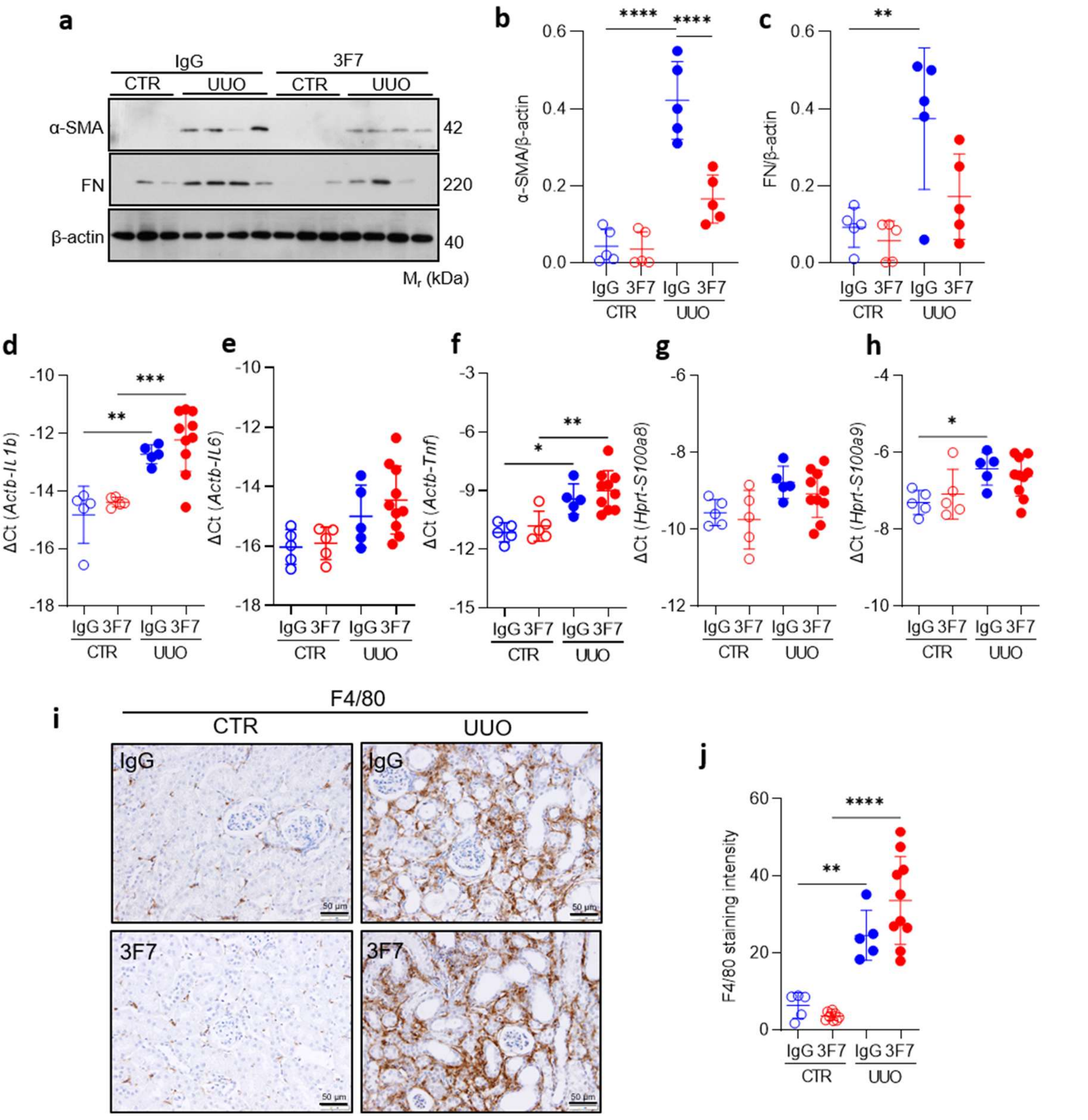

Supplementary Figure 2. Inhibition of FXIIa does not affect macrophage numbers and expression of inflammatory mediators in UUO kidneys at day 10 post ligation. a) Representative western blots showing α-SMA and FN levels in UUO and CTR kidneys of animals injected either with IgG or 3F7. Β-actin was used as a loading control. b, c) Densitometric analysis of western blots depicted in (a). n=5/group. d-h) mRNA expression of *Il1b* (d), *Il6* (e), *Tnfa* (f), *S100a8* (g), and *S100a9* (h) in UUO and CTR kidneys of mice treated either with IgG or 3F7. i) F4/80 staining in representative sections of UUO and CTR kidneys of mice treated either with IgG or 3F7. Bar 50 µm. j) F4/80 staining quantification. All samples were obtained at day 10 post ligation. n=5-10/group. ****p<0.0001; ***p<0.001; **p<0.01; *p<0.05.

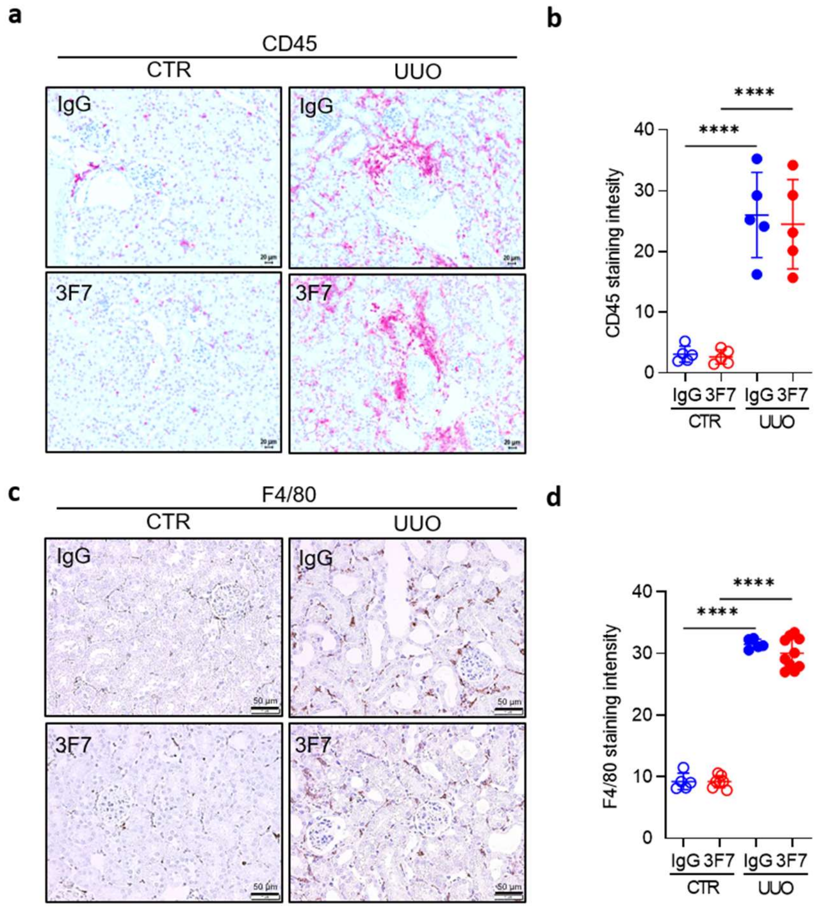

Supplementary Figure 3. Inhibition of FXIIa does not alter CD45+ cell and F4/80 macrophage numbers in UUO kidneys at day 3 post ligation. a, c) CD45 (a) and F4/80 (c) staining in representative kidney sections of UUO and CTR mice treated either with IgG or 3F7. The animals were sacrificed at day 3 post ligation. Bar: 20 µm (a) and 50 µm (c). (b, d) CD45 (b) and F4/80 (d) staining quantification. n=5-10/group. ****p<0.0001.

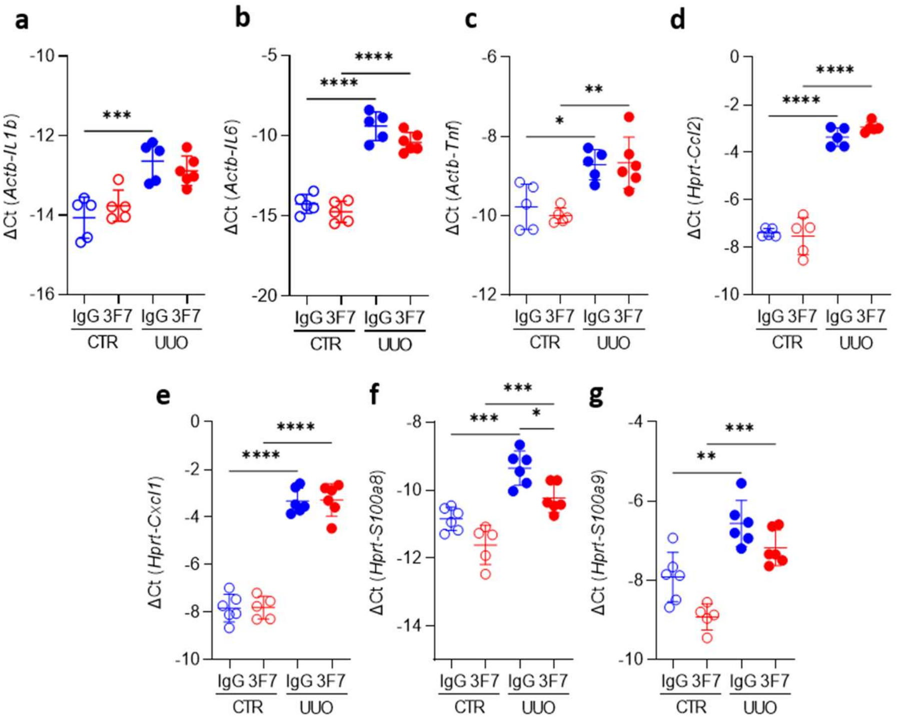

Supplementary Figure 4. Inhibition of FXIIa decreases mRNA expression of S100a and S100a9 in obstructed kidneys at day 3 post 3F7 administration. a-g) mRNA expression of *Il1b* (a), *Il6* (b), *Tnfa* (c), *Ccl2* (d), *Cxcl1* (e), *S100a8* (f), and *S100a9* (g) in UUO and CTR kidneys of mice treated either with IgG or 3F7 for 3 days. The qPCR data are presented as a ΔCt using *Actb* (β-actin) or *Hprt* (hypoxanthine guanine phosphoribosyltransferase) as a reference gene. n=5-6/group. ****$p<0.0001$; ***$p<0.001$; **$p<0.01$; *$p<0.05$.

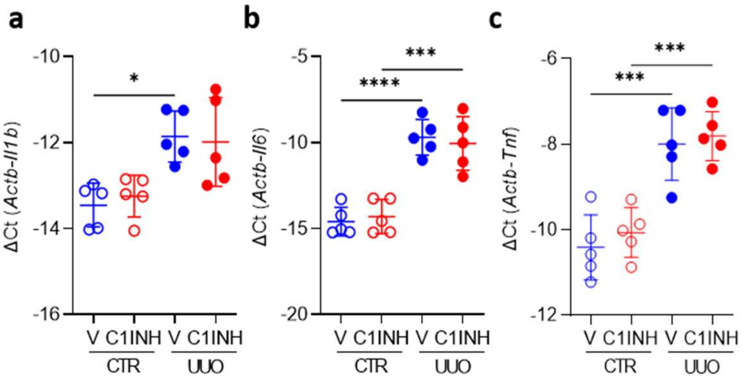

Supplementary Figure 5. C1 Esterase Inhibitor administration does not alter expression of the inflammatory mediators in UUO-induced kidney injury model. a-c) mRNA expression of *Il1b* (a), *Il6* (b), and *Tnfa* (c) in UUO and CTR kidneys of mice treated either with Vehicle (V) or C1 Esterase Inhibitor (C1INH). The animals were sacrificed at day 3 post ligation. The qPCR data are presented as a ΔCt using *Actb* (β-actin) as a reference gene. n=5/group. ****$p<0.0001$; ***$p<0.001$; *$p<0.05$.

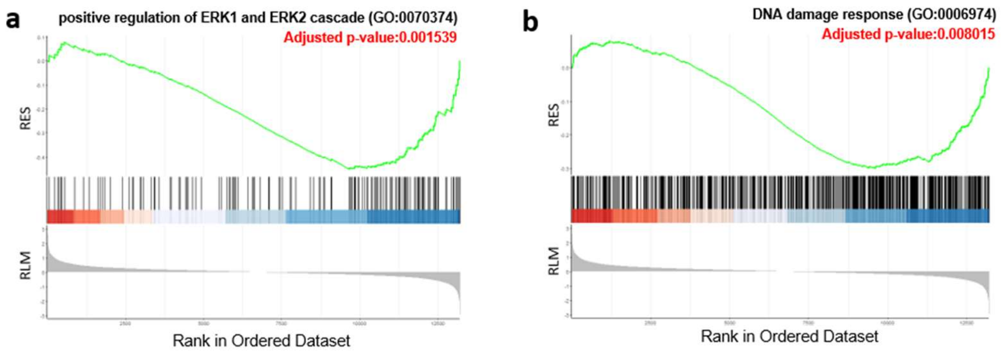

Supplementary Figure 6. Microarray data of unilateral ureteral obstruction (UUO) kidney samples confirm the efficacy of 3F7 in reducing profibrotic and stress-mediated signaling pathways. a, b) Positive regulation of ERK1 and ERK2 cascade (a) and DNA damage response (b) related gene expression is downregulated upon 3F7 treatment in Gene Set Enrichment Analysis (GSEA). The x-axis displays gene ranks based on log2 fold change. RLM = Ranked List Metric, displaying gene expression fold change. RES = Running Enrichment Score, indicated by the green line.

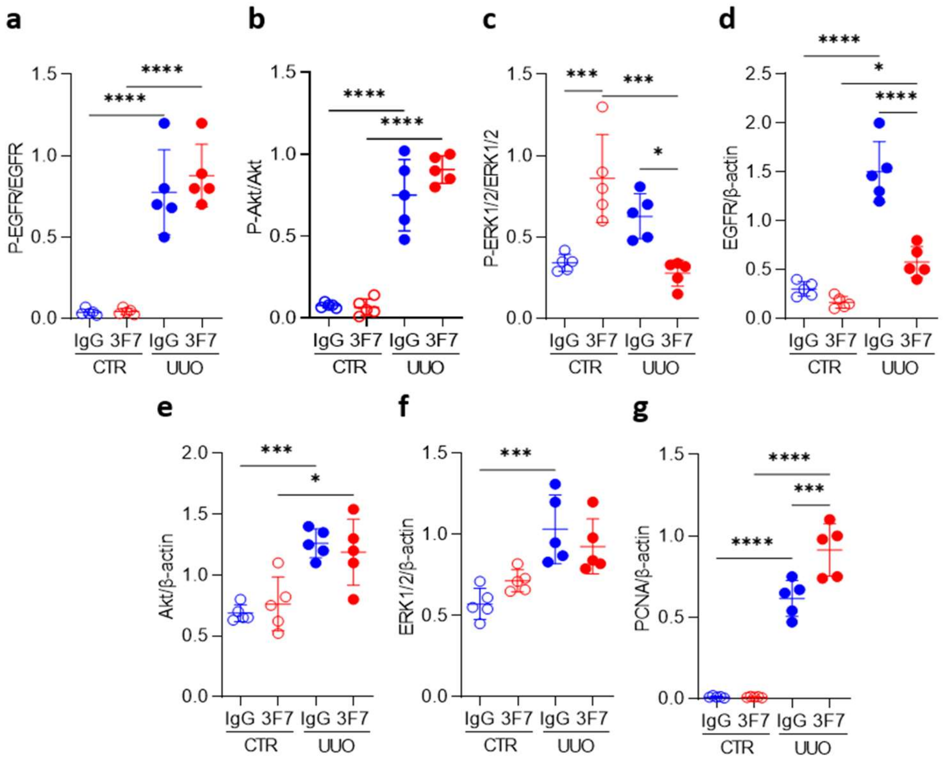

Supplementary Figure 7. Treatment with 3F7 decreases the activity of ERK1/2 and the expression of EGFR, but increases the expression of PCNA in UUO kidneys compared with the UUO IgG group. a-g) Densitometric analysis of western blots depicted in Figure 5a of the main manuscript. n=5/group. ****p<0.0001; ***p<0.001; *p<0.05.
